# Tendon Transfer to Treat Radial Nerve Palsy Following COVID-19 Infection

**DOI:** 10.7759/cureus.49595

**Published:** 2023-11-28

**Authors:** Sydney A Thai, Anirudh Kulkarni, Ajul Shah

**Affiliations:** 1 Orthopedic Surgery, Robert Wood Johnson Medical School, New Brunswick, USA; 2 Plastic Surgery, Center for Hand and Upper Extremity Surgery, Shrewsbury, USA

**Keywords:** plastic and reconstructive surgery, ortho surgery, mono-neuropathy, covid 19, tendon graft

## Abstract

Multiple manifestations have been associated with the novel severe acute respiratory syndrome coronavirus 2 (SARS-CoV-2) virus. Among them are mononeuritis multiplex (MNM) and other neurological complications, whose connection to coronavirus disease 2019 (COVID-19) is still unclear. One of the most common sites of nerve injury is the radial nerve, which can be treated with both nerve or tendon transfer. In this case report, a patient who was afflicted with severe COVID-19 infection and developed mono neuritis multiplex after prolonged mechanical ventilation with radial nerve palsy was treated with multiple tendon transfers. This is a way to use an established mechanism of resolving the manifestations of radial nerve palsy to aid in the recovery of COVID-19-related mononeuritis multiplex.

## Introduction

The novel severe acute respiratory syndrome coronavirus 2 (SARS-CoV-2) causing coronavirus disease 2019 (COVID-19) has placed an enormous strain on healthcare systems across the world, resulting in almost seven million deaths since its discovery [1]. COVID-19 has a number of severe respiratory manifestations, but in the setting of prolonged intensive care unit (ICU) stays with mechanical ventilation, patients have also demonstrated focal neurological deficits, with central and peripheral nervous system manifestations, namely Guillain-Barre syndrome and mononeuritis multiplex (MNM) [2-5].

MNM is defined as a peripheral neuropathy involving two or more noncontiguous nerves, usually presenting as motor or sensory symptoms in an asymmetric pattern [6]. A common site of nerve dysfunction in the setting of COVID-19 is the radial nerve, resulting in radial nerve palsy [7,8]. Radial nerve palsy results in a loss of wrist extension, thumb extension, and finger extension, leading to a severe loss of function in the upper extremities; traditionally, there are several available treatment options [4,7,8]. Nerve transfers are an option to restore radial nerve function with positive outcomes. Branches of the median nerve are often used, and the donor nerve must: 1) have an expendable function that will act synergistically with the recipient; 2) have no sensory fibers and sufficient axon counts; 3) be close to the recipient nerve and motor endplates. Disadvantages of nerve transfers include long recovery time and its time-sensitive nature since the motor endplate degenerates [9].

In cases where a nerve transfer may not be possible, such as the extensive nerve damage seen in MNM, tendon transfers have a profound palliative effect in treating radial nerve palsy [9]. The candidate must meet several requirements, namely: 1) the hand and wrist joints must be supple and have a passive range of motion (ROM); 2) the donor muscle and tendon unit must be expendable and have adequate strength and excursion; 3) the donor muscle/tendon must have a straight line of pull and repair only one function; 4) the unit must have synergistic function [9,10]. Complications include altered biomechanics, scar formation, and prolonged stiffness or immobilization [9].

We present the case of a patient who suffered from radial nerve palsy following MNM after COVID-19 infection, using an established tendon transfer technique to aid in recovery.

## Case presentation

We present the case of a 49-year-old male who was afflicted with a severe COVID-19 infection in November 2021, requiring intubation and prolonged ICU care, including mechanical ventilation and tracheostomy, and awoke in December 2021. His past medical history was notable for hypertension, pre-diabetes, cervical spinal decompression seven years prior, nephrostomy, testicular cancer with metastases in the abdomen, and obesity. Upon being weaned from sedation, there was a marked loss of upper extremity function bilaterally, which was addressed through rehabilitation following discharge.

In March 2022, the patient was seen by Neurology and had an electromyography (EMG) study performed confirming MNM. On physical examination, the left upper extremity demonstrated a marked decrease in function overall with the absence of triceps, wrist and finger, and thumb extensor function. Notably, radial sensory nerve sensation was completely deficient. The remainder of the examination demonstrated the functionality of the extremity distal to the elbow but with weakness of muscles innervated by the median and ulnar nerves and hypoactive deep tendon reflexes.

In June 2022, the patient had repeat studies done which showed MNM with clinical and electrodiagnostic evidence of severe left radial dysfunction as well as moderate bilateral carpal tunnel syndrome. Electrodiagnostic findings showed diminished function of the median nerve, including prolonged median terminal motor latencies, markedly low amplitude left radial motor responses, slow left median motor conduction velocity, and prolonged low amplitude median nerve sensory action potentials. The left radial sensory nerve action potentials were completely absent.

Needle EMG examination of the relevant muscles of the bilateral upper extremities in the C5-T1 myotomal distribution revealed active denervation in the left triceps, extensor dig communis, and extensor indicis muscles. There was an absence of motor unit potentials in the left triceps and decreased recruitment in all other sampled muscles of the upper extremities. There was an unchanged, persistent, severe lesion of the left radial nerve.

After discussion with the patient, it was determined that intraoperative evaluation and neurolysis of the radial nerve would be performed with neurolysis at the spiral groove and radial tunnel, and if no functionality returned, then tendon transfers would be performed. Tendon transfers were chosen over nerve transfers due to the concern for MNM affecting the donor's nerves, potentially compromising their function. Furthermore, the patient had been suffering from significant loss of upper extremity function for twelve months prior to reconstruction.

After decompression and neurolysis of the radial nerve, no functionality returned. Therefore, the following tendon transfers were performed: 1) Left flexor carpi radialis to extensor digitorum communis to index, middle, ring, and small fingers; 2) Left flexor digitorum superficialis to the fourth finger to left extensor pollicis longus, as the patient had an absent palmaris longus tendon; 3) Left pronator teres to extensor carpi radialis brevis.

The first objective was to isolate the flexor carpi radialis (FCR) tendon, which was dissected circumferentially to the wrist crease. The pronator teres tendon (PT) was also identified following a deeper dissection and was incised from the radius along with a portion of the periosteum. The initial approach was also to find the palmaris longus tendon (PL), however, upon the dissection on the ulnar side, no functioning PL was identified. Instead, the flexor digitalis superficialis (FDS) of the fourth finger were harvested for use, as described by Cheah et al. [10].

The decision was made upon the final identification of the recipient's tendons to perform end-to-end transfers rather than side transfers. The use of the PT as a donor and transferring it to the ECRB is a reliable method to restore wrist extension [7] and was achieved by securing the PT to the ECRB using multiple 2-0 Ethibond sutures with the wrist in complete extension, but while ensuring appropriate tension and preventing overtightening. To restore thumb extension, the FDS of the fourth finger of the left hand was first attached to the EPL via a Pulvertaft weave and secured using the 2-0 Ethibond sutures. The harvested FCR was then brought towards the extensor digitorum communis (EDC) of each of the four digits, and secured using multiple Pulvertaft weaves and 2-0 Ethibond sutures.

The patient was immobilized for six weeks post-operatively and then enrolled in an occupational hand therapy program. At the nine-month follow-up, the patient’s functionality was significantly improved with the activation of each of the tendon transfers, and he could utilize the left upper extremity for multiple activities in his daily life (Supplemental Video 1).

**Video 1:** Nine-month post-operative follow-up progress report FCR: flexor carpi radialis; EDC: extensor digitorum carpi; FDS: flexor digitorum superficialis; EPL: extensor pollicus longus; PT: pronator teres; ECRB: extensor carpi radialis brevis.

## Discussion

COVID-19 has been linked to a variety of manifestations, with a number of patients presenting with neurological complications [8]. In those who require prolonged mechanical ventilation and ICU stay, prone positioning has been shown to improve oxygenation and survival rates [3]. However, those who undergo prolonged prone positioning and mechanical ventilation can develop focal neurological deficits resembling MNM. It has been suggested that such nerve injuries develop during the period of ventilation [4].

There have been several theories proposed in the literature to describe the underlying process of nerve damage following COVID-19 infection. Some proposed mechanisms of the neuritis arising post infection involve the damage caused by a dramatic increase in interleukin (IL)-1, IL-6, and tumor necrosis factor (TNF) classified as the “cytokine storm” which leads to a profound overactivation of host immune cells leading to systemic damage, though a clear link to peripheral nervous system damage has not been established [11]. Molecular mimicry has also been explored as a potential explanation for the myelitis and encephalitis commonly observed after COVID-19 infection after isolation of anti-neuronal antibodies was observed in patients [11]. While plausible, as there has been some support for cross-reactivity between antigens targeting COVID-19 antigens and host cells, the theory of molecular mimicry is unlikely, with little homology being observed between the axonal or neural surface proteins and the COVID-19 proteins studied thus far.

A prothrombotic state is another potential mechanism leading to nerve damage. The large and small vessel damage that arises following large-scale thrombosis may lead to ischemia of the vasa nervorum leading to diffuse neuritis [2]. An exploration of the prone position used to manage Acute Respiratory Distress Syndrome (ARDS) has elucidated the potential complication of nerve damage in patients, especially those having undergone severe disease requiring time in the ICU. The position of patients in this setting has led to the observation of entrapment neuropathies, with the ulnar nerve and brachial plexus being impacted most commonly [11]. While there may not be a singular mechanism identified explaining the nerve damage that has been observed in patients after COVID-19 infection, they underline the importance of addressing the sequelae of neuritis during recovery.

There have been a few studies that compare nerve and tendon transfer outcomes, but Patterson et al. showed that both result in improvements in quality of life (QOL) and Disabilities of the Arm, Shoulder, and Hand (DASH) scores [9]. Additionally, the nerve transfer cohort showed significantly greater grip strength postoperatively, while the tendon transfer cohort saw faster recovery times. The length of follow-up was longer in nerve transfer patients to allow for reinnervation, and the patients who underwent nerve transfers anecdotally illustrated independent finger extension and dexterity; as such, it has been suggested that nerve transfers are best for younger patients who have activity expectations of finger dexterity. On the other hand, patients who present later and who require a shorter recovery time are recommended to undergo tendon transfers [9].

While a nerve transfer in the context of radial nerve injury would have been an acceptable treatment, our patient demonstrated concern for diminished functionality of the donor nerves, as evidenced by physical examination and electrodiagnostic studies. The transfer would have been possible, but there was a risk of failure to achieve appropriate function considering the effect of the MNM on the donor nerves as well as the time elapsed from the onset of radial nerve paralysis. Rather, tendon transfer was preferred to proceed and resolve the radial nerve palsy.

Following proper assessment of the tension of each of the three major transfers, and completion of the procedure, the patient was continuously followed and enrolled in a hand therapy program. Over the course of the last nine months, the patient demonstrated excellent recovery of wrist extension, thumb extension, as well as digit extension in each of the digits in the left hand. The patient thus far has had positive outcomes, and follow-up will continue over time to assess longitudinal outcomes.

While there were initial concerns that the patient would not be a candidate for the tendon transfers due to his muscular strength deficiencies as related to COVID-19, the team understood this trepidation and ultimately decided to move forward to give him the best chance at recovery. Our objective is to demonstrate that tendon transfers can be done in the face of MNM and COVID-19 paralysis, a condition that is surprisingly frequent and can result in severe disability [5,11]. We focus on the treatment of an already established palsy; however, in future considerations, prevention of such injuries would be the primary goal, perhaps with prolonged prone positioning in an intensive care setting to protect the cervical nerves. Ultimately, this method can be used to aid patients in recovering from the virus and improving their quality of life [5].

## Conclusions

COVID-19 remains a constantly evolving virus with numerous sequelae with devastating effects, as well as an emerging pattern of nerve damage following MNM. Diffuse neural damage can be addressed through both nerve or tendon transfers. While nerve transfers are possible, tendon transfers have been proven to also be a robust option to explore in patients with insufficient neural donors due to previous complications or, as in the case of MNM, diffuse nerve damage limiting proper nerve function. While this was a successful treatment for this case, prevention is still the best option, with proper positioning in intensive care settings to limit injury to the nerves. As reported within this case and its outcomes, COVID-19-induced paralysis of the radial nerve can successfully be resolved using tendon transfers and should be explored further to resolve other issues brought upon by mononeuritis multiplex.
